# Aquaporins in the Colon as a New Therapeutic Target in Diarrhea and Constipation

**DOI:** 10.3390/ijms17071172

**Published:** 2016-07-20

**Authors:** Nobutomo Ikarashi, Risako Kon, Kiyoshi Sugiyama

**Affiliations:** Department of Clinical Pharmacokinetics, Hoshi University, 2-4-41 Ebara, Shinagawa-ku, Tokyo 142-8501, Japan; r-kon@hoshi.ac.jp (R.K.); sugiyama@hoshi.ac.jp (K.S.)

**Keywords:** aquaporin, colon, diarrhea, laxative, constipation, morphine

## Abstract

Aquaporins (AQPs) play important roles in the water transport system in the human body. There are currently 13 types of AQP, AQP0 through AQP12, which are expressed in various organs. Many members of the AQP family are expressed in the intestinal tract. AQP3 is predominantly expressed in the colon, ultimately controlling the water transport. Recently, it was clarified that several laxatives exhibit a laxative effect by changing the AQP3 expression level in the colon. In addition, it was revealed that morphine causes severe constipation by increasing the AQP3 expression level in the colon. These findings have shown that AQP3 is one of the most important functional molecules in water transport in the colon. This review will focus on the physiological and pathological roles of AQP3 in the colon, and discuss clinical applications of colon AQP3.

## 1. Introduction

Constipation and diarrhea are common clinical complaints that negatively affect quality of life. In recent years, the number of patients with constipation has been rapidly increasing due to the Westernization of dietary patterns and the aging society [1]. In palliative care, many patients are taking morphine for pain control, and almost all of these patients suffer from constipation [2,3]. Although they have received symptomatic therapies using laxatives, an adequate therapeutic effect is not always achieved. Therefore, it is necessary to develop a new strategy of constipation. On the other hand, Crohn’s disease and ulcerative colitis patients who have severe diarrhea have also been increasing [4]. In the rapidly increasing elderly population, drug-induced diarrhea in the elderly is one of the problems for drug therapy [5]. Therefore, it is important to perform appropriate treatment after clarified the diarrhea mechanism.

Recently, it has become clear that aquaporins (AQPs) play important roles in the water transport system in the human body [6]. AQPs are water channels through which water and glycerol are selectively transported. There are currently 13 types of AQP, AQP0 through AQP12, which are expressed in various organs [7,8,9,10]. Many members of the AQP family are expressed in the intestinal tract: AQP1, AQP3, AQP4, AQP7, AQP8, AQP9, and AQP10 are expressed in the colon, which ultimately controls fecal water content [11,12,13,14,15,16,17,18,19,20,21]. In human colon, AQP3 is predominantly expressed in mucosal epithelial cells [15,19]. Therefore, it is believed that AQP3 plays an important role in water transport in the colon. However, the physiological role and the regulation of AQP3 expression are little known. It is considered that analysis of AQP3 in the colon might lead to the development of new treatments and a prevention method for constipation and diarrhea. This review will provide an overview of the role of colon AQP3 under physiological and pathophysiological conditions, as well as clinical applications involving AQP3. 

## 2. Localization of AQP3 in the Colon

In the mucosal epithelial cells in mice colon, AQP4 is predominantly expressed. Wang et al. reported that fecal water content increases in AQP4 knockout mice relative to wild-type mice [22]. This result suggests that AQP expression in the mucosal epithelial cells in the colon is one important factor that controls the water content of feces.

**Figure 1:**
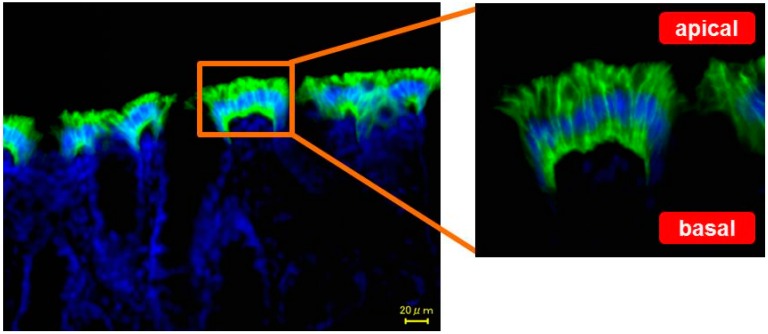
Distribution of aquaporin-3 (AQP3) expression in the rat colon.

In human colon, the expression level of AQP4 is low, while AQP3 is predominantly expressed in the mucosal epithelial cells [23]. Therefore, there are many reports about AQP3 in the colon [12,15,19,24]. Many reports have discussed the intracellular localization of AQP3. First, Silberstein et al. reported that AQP3 was strongly expressed at the apical side of mucosal epithelial cells in human colon, while its expression level at the basolateral side was low [15]. Subsequently, Mobasheri et al. reported that AQP3 was present at the basolateral side [19]. In addition, Rai et al. clarified that NH_2_-terminal sorting signal mediates the basolateral targeting of AQP3 [25]. It was also reported that AQP7 and AQP8 were localized at the apical side and AQP3 was localized at the basolateral side [26,27,28]. On the other hand, it was clarified that AQP3 is predominantly expressed at both the apical and basal sides of mucosal epithelial cells in rat colon (Figure 1) [29]. 

## 3. Relation between AQP3 Expression and Diarrhea

Yamamoto et al. revealed that allergic diarrhea is associated with a downregulation in AQP4 and AQP8 in the colon [30]. It was also reported that AQP1, AQP3, and AQP11 were decreased in the colon of Crohn’s disease and ulcerative colitis patients [21]. When diarrhea occurred after small bowel resection and gradually improves due to intestinal adaptation, AQP3 in the colon were up-regulated during adaptation [31]. In previous studies, it has been reported that a gastrointestinal hormone such as vasoactive intestinal polypeptide (VIP) caused Verner–Morrison syndrome, which is associated with diarrhea [32]; diarrhea occurs after the intravenous administration of VIP to healthy individuals [33]; and AQP3 expression levels increase after VIP treatment in HT-29 cells derived from human colon cancer [24]. Based on these reports, it is considered that AQP3 plays an important role in water transport in the colon. 

### 3.1. Role of AQP3 in the Colon in the Laxative Effect of Magnesium Sulfate

**Figure 2:**
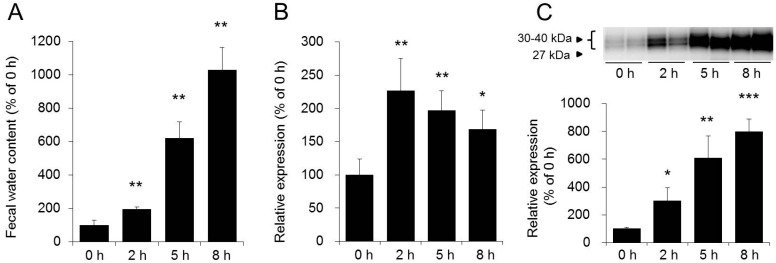
Effect of magnesium sulfate on fecal water content (**A**); the mRNA expression level of sodium *myo*-inositol transporter, gene associated with osmotic pressure (**B**); and AQP3 protein expression level (**C**) in the rat colon. Dunnett’s test: * *p* < 0.05, ** *p* < 0.01, and *** *p* < 0.001 vs. 0 h. Adapted with permission from Ikarashi et al. [29]. Copyright 2011 The Pharmaceutical Society of Japan.

**Figure 3:**
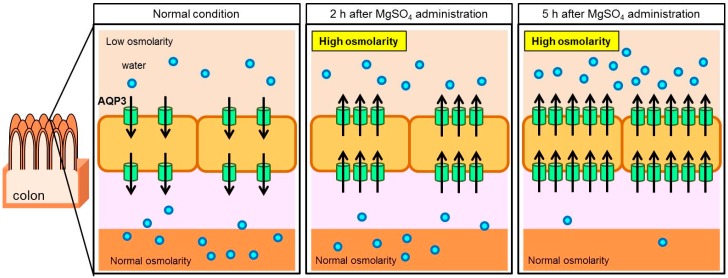
Water transport in the colon after magnesium sulfate administration.

It is believed that osmotic laxatives, such as magnesium sulfate, induce diarrhea by causing an increase in the osmotic pressure in the intestinal tract [34]. After oral magnesium sulfate administration to rats, fecal water content and the AQP3 expression level in the colon increased significantly in time-dependent manner. These changes in AQP3 expression level correlated well with the changes in fecal water content. On the other hand, osmotic pressure in the colon decreased with time from the peak level observed at two hours after administration (Figure 2) [29]. Based on the above results, the laxative effect of magnesium sulfate was considered to be exhibited via the following mechanism. Under physiological conditions, water is transported from the luminal side, where the osmotic pressure is low, to the vascular side, where the osmotic pressure is high, via AQP3. Water is transported from the vascular side to the luminal side after the administration of magnesium sulfate, because the osmotic pressure in the lumen of the colon has risen. At two hours after the administration, a large amount of water was not transported, because the AQP3 expression level was not sufficiently elevated. However, at subsequent time points, the AQP3 expression level significantly increased, which caused the transport of a large amount of water to the luminal side, resulting in the occurrence of diarrhea (Figure 3). Based on these findings, the laxative effect of magnesium sulfate is not simply caused by a change in the osmotic pressure in the intestinal tract, but could be a response to increased AQP3 expression.

The mechanism by which magnesium sulfate increased the AQP3 expression level was revealed that an increase in the intracellular Mg^2+^ concentration may trigger cAMP response element binding protein (CREB) phosphorylation through protein kinase A activation, and promote AQP3 gene transcription [35].

### 3.2. Role of AQP3 in the Colon in the Laxative Effects of Bisacodyl and Sennoside A

**Figure 4:**
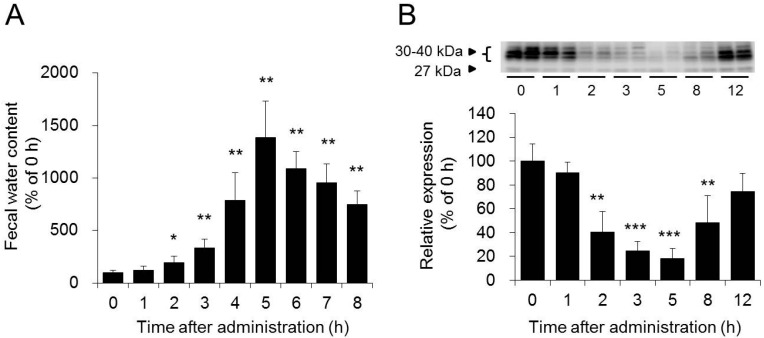
Effect of bisacodyl on fecal water content (**A**); and AQP3 protein expression level in the rat colon (**B**). Dunnett’s test: * *p* < 0.05, ** *p* < 0.01, and *** *p* < 0.001 vs. 0 h. Adapted with permission from Ikarashi et al. [38]. Copyright 2011 The American Physiological Society.

Bisacodyl, which is classified as a stimulant laxative, exhibits its laxative effect by enhancing the peristaltic movements of the bowel [36,37]. After oral administration of bisacodyl to rats, unlike magnesium sulfate, bisacodyl caused severe diarrhea without changing the osmotic pressure inside the colon. The expression level of AQP3 decreased significantly from two hours after the administration, and a good correlation was observed between this decrease and the increase in fecal water content (Figure 4) [38]. Experiments using AQP3 inhibitors such as mercury chloride [39] and copper sulfate [40] showed that diarrhea was induced when the AQP3 activity in the colon was inhibited, without changing the osmotic pressure of the intestinal tract [41]. These results suggest that laxative effect of bisacodyl might be attributable to the decrease in the AQP3 expression level. Briefly, bisacodyl decreases AQP3 expression level in the colon, and causes a decrease in water transport from the luminal side to the vascular side, resulting in exhibiting its laxative effect.

Previous studies showed that bisacodyl activates macrophages in the colon [36,42]; that this activation induces the secretion of inflammatory cytokines and prostaglandin E_2_ (PGE_2_) via an increase in the expression of cyclooxygenase-2 (COX-2) [43,44]; and that tumor necrosis factor-α (TNF-α) [45,46,47] and PGE_2_ [48,49] decrease the expression level of AQP. Accordingly, it has become clear that bisacodyl activates directly colon macrophage, and increases the secretion of PGE_2_, which acts as a paracrine factor and decreases AQP3 expression in colon mucosal epithelial cells [38]. In addition, it was revealed that sennoside A, which is classified as a stimulant laxative, also exhibits a laxative effect by decreasing the expression level of AQP3 in the colon via a mechanism similar to bisacodyl [50]. It was also shown that pre-administration of indomethacin such as a COX inhibitor to rats suppressed the secretion of PGE_2_, resulting in the suppression of the laxative effect of bisacodyl and sennoside A and the decrease in the expression level of AQP3. 

## 4. Relation between AQP3 Expression and Constipation

AQP3 in the colon of rat models with slow transit constipation was down-regulated and AQP4 and AQP8 were not changed [51]. In addition, it was reported that AQP9 in the colon of patients with slow transit constipation was increased [52]. To date, little is known about the relation between AQP and constipation. 

Morphine is a narcotic analgesic that has high potency but causes severe constipation as an adverse effect [2,3]. Morphine suppresses the peristaltic movements of the bowel, resulting in the development of constipation [53]. However, other mechanisms such as water transport in the colon have been poorly understood. After the oral administration of morphine to rats, constipation was induced and the expression level of AQP3 significantly increased. HgCl_2_ improved in the symptoms of morphine-induced constipation [54]. Based on these results, it is suggested that morphine increases the expression level of AQP3 in the colon, which enhances the water transport from the luminal side to vascular side, resulting in hardening of the feces.

**Figure 5:**
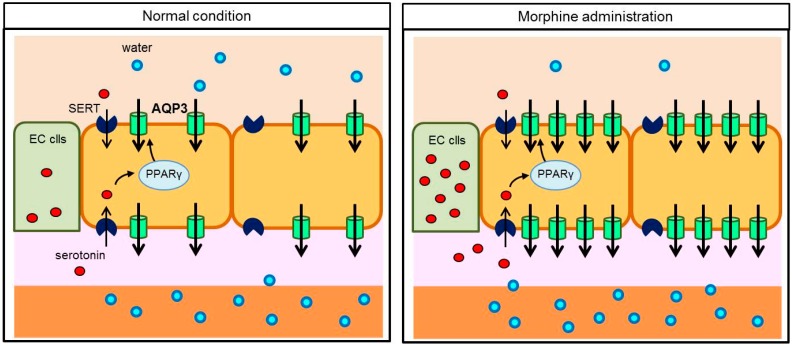
Water transport in the colon after morphine administration.

It was has been reported that morphine stimulates the release of serotonin from the intestinal wall and suppresses peristaltic movements [55]. There is a large amount of serotonin in EC cells in the intestinal tract [56]. Serotonin secreted from EC cells is metabolized after being taken into the cells by serotonin reuptake transporter (SERT) [57]. Serotonin is a ligand for peroxisome proliferator-activated receptor gamma (PPARγ), nuclear receptor, which contributes to epithelial cell proliferation and turnover [58]. In contrast, PPARγ agonists increase the AQP3 expression level [59]. Accordingly, morphine-induced serotonin secreted from the colon was taken into cells by SERT and activated PPARγ, which subsequently increased AQP3 expression levels (Figure 5) [54].

## 5. AQP and Clinical Application

### 5.1. Role of AQP3 in the Colon in the Concomitant Use of Laxatives

**Figure 6:**
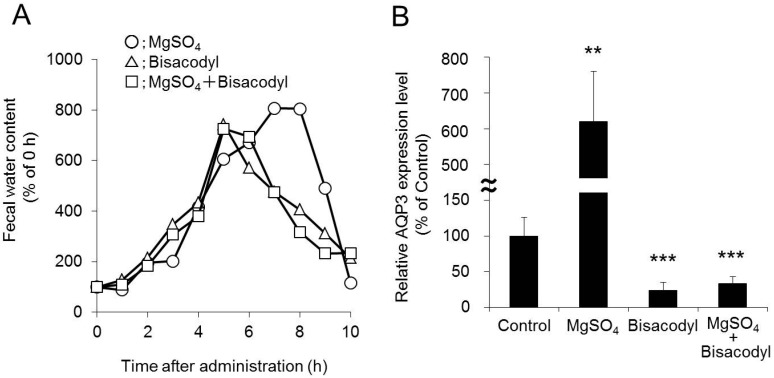
Effect of combination of magnesium sulfate and bisacodyl on fecal water content (**A**); and AQP3 protein expression in the rat colon (**B**). Dunnett’s test: ** *p* < 0.01, and *** *p* < 0.001 vs. control group. Adapted with permission from Ikarashi et al. [60]. Copyright 2012 Elsevier.

In a clinical practice, an osmotic laxative is prescribed as the first-line drug for treatment of patients with severe constipation, and if it is not effective, other laxatives with different mechanisms of action, including stimulant laxatives, are concomitantly used. However, since there is no clear evidence that these enhance the laxative effects by concomitant use of different types of laxatives, patients are currently receiving empirically-based treatment. When magnesium sulfate and bisacodyl were concomitantly administered to rats, the observed laxative effect was lower than that observed after administration of magnesium sulfate alone, and similar to that observed after administration of bisacodyl alone. The fact was considered to be the reason that the expression pattern of AQP3 in the colon after the concomitant administration was very similar to that after bisacodyl administration alone (Figure 6) [60]. 

The above-mentioned results clearly show that the concomitant administration of different types of laxatives does not always lead to an enhanced laxative effect. Currently, multiple laxatives are used concomitantly in patients with severe constipation, without definite evidence of efficacy. The increase in the number of drugs leads to an increase in drug–drug interactions. In the future, it is necessary that the evidence supporting the therapeutic efficacy of laxatives be clearly identified to facilitate the proper use of laxatives.

### 5.2. Efficacy of Laxatives on Morphine-Induced Constipation and AQP3 in the Colon

Previously, morphine was considered to induce constipation by suppressing the peristaltic movements of the bowel [55]. However, in many cases, it is difficult to treat morphine-induced constipation, even with the use of stimulant laxatives such as sennoside A and bisacodyl. Based on the previous findings [53], it was considered that these laxatives have no effect for morphine-induced constipation for the following reasons. The treatment of pain in cancer patients is managed according to the “WHO three-step analgesic ladder”, and a good analgesic effect is achieved by the concomitant use of morphine and non-steroidal anti-inflammatory drugs (NSAIDs). However, it has become clear that the laxative effect of bisacodyl and sennoside A cannot be exhibited when NSAIDs are concomitantly used [38,50]. It is considered to be one of the reasons why these laxatives are not effective in the treatment of morphine-induced constipation. Therefore, for the treatment of constipation in those patients who have to take NSAIDs in cancer pain relief, a laxative that is not affected by NSAIDs, such as a prostaglandin drugs, may improve the symptoms of constipation by lowering the expression level of AQP3 in the colon. As mentioned above, it is necessary to analyze the laxative effect for evidenced-based medicine. 

## 6. Conclusions

Based on the above results, it has become clear that the expression level of AQP3 in the colon plays an important role in the laxative effects by osmotic laxatives and stimulant laxatives. It was also shown that an increase in the expression level of AQP3 is involved in onset of morphine-induced constipation. Researchers should analyze the relation between AQP and constipation using other constipation model because little is reported about this point. Although it is likely that AQP3 expressed at both the apical and basal sides of mucosal epithelial cells in rat colon (Figure 1), there is the possibility that key molecules of apical side in the colon are AQP7 and AQP8 [26,27,28]. Numerous discussions are still underway regarding the intracellular localization of AQP3 in the intestinal tract. By continuing efforts to examine the expression and functions of AQP other than AQP3 in the intestinal tract and investigating the mechanism of water transport, new laxatives and antidiarrheal drugs targeting AQP might be developed in the future.

## Abbreviations

AQP	aquaporin
CREB	cAMP response element binding protein
PGE_2_	prostaglandin E_2_
COX-2	cyclooxygenase-2
TNF-α	tumor necrosis factor-α
SERT	serotonin reuptake transporter
PPARγ	peroxisome proliferator-activated receptor gamma
NSAIDs	non-steroidal anti-inflammatory drugs

## References

[1] Hall K.E., Proctor D.D., Fisher L., Rose S. (2005). American gastroenterological association future trends committee report: Effects of aging of the population on gastroenterology practice, education, and research. Gastroenterology.

[2] Quigley C. (2005). The role of opioids in cancer pain. BMJ.

[3] Dal Molin A., McMillan S.C., Zenerino F., Rattone V., Grubich S., Guazzini A., Rasero L. (2012). Validity and reliability of the italian constipation assessment scale. Int. J. Palliat. Nurs..

[4] Molodecky N.A., Soon I.S., Rabi D.M., Ghali W.A., Ferris M., Chernoff G., Benchimol E.I., Panaccione R., Ghosh S., Barkema H.W. (2012). Increasing incidence and prevalence of the inflammatory bowel diseases with time, based on systematic review. Gastroenterology.

[5] Jain V., Pitchumoni C.S. (2009). Gastrointestinal side effects of prescription medications in the older adult. J. Clin. Gastroenterol..

[6] Loo D.D., Wright E.M., Zeuthen T. (2002). Water pumps. J. Physiol..

[7] Agre P. (2004). Aquaporin water channels (Nobel Lecture). Angew. Chem. Int. Ed. Engl..

[8] King L.S., Kozono D., Agre P. (2004). From structure to disease: The evolving tale of aquaporin biology. Nat. Rev. Mol. Cell Biol..

[9] Hara-Chikuma M., Verkman A.S. (2006). Physiological roles of glycerol-transporting aquaporins: The aquaglyceroporins. Cell. Mol. Life Sci..

[10] Ishibashi K., Tanaka Y., Morishita Y. (2014). The role of mammalian superaquaporins inside the cell. Biochim. Biophys. Acta.

[11] Hasegawa H., Lian S.C., Finkbeiner W.E., Verkman A.S. (1994). Extrarenal tissue distribution of CHIP28 water channels by in situ hybridization and antibody staining. Am. J. Physiol..

[12] Ishibashi K., Sasaki S., Saito F., Ikeuchi T., Marumo F. (1995). Structure and chromosomal localization of a human water channel (AQP3) gene. Genomics.

[13] Koyama N., Ishibashi K., Kuwahara M., Inase N., Ichioka M., Sasaki S., Marumo F. (1998). Cloning and functional expression of human aquaporin8 cDNA and analysis of its gene. Genomics.

[14] Koyama Y., Yamamoto T., Tani T., Nihei K., Kondo D., Funaki H., Yaoita E., Kawasaki K., Sato N., Hatakeyama K. (1999). Expression and localization of aquaporins in rat gastrointestinal tract. Am. J. Physiol..

[15] Silberstein C., Kierbel A., Amodeo G., Zotta E., Bigi F., Berkowski D., Ibarra C. (1999). Functional characterization and localization of AQP3 in the human colon. Braz. J. Med. Biol. Res..

[16] Gallardo P., Cid L.P., Vio C.P., Sepulveda F.V. (2001). Aquaporin-2, a regulated water channel, is expressed in apical membranes of rat distal colon epithelium. Am. J. Physiol. Gastrointest. Liver Physiol..

[17] Hardin J.A., Wallace L.E., Wong J.F., O’Loughlin E.V., Urbanski S.J., Gall D.G., MacNaughton W.K., Beck P.L. (2004). Aquaporin expression is downregulated in a murine model of colitis and in patients with ulcerative colitis, Crohn’s disease and infectious colitis. Cell Tissue Res..

[18] Matsuzaki T., Tajika Y., Ablimit A., Aoki T., Hagiwara H., Takata K. (2004). Aquaporins in the digestive system. Med. Electron. Microsc..

[19] Mobasheri A., Wray S., Marples D. (2005). Distribution of AQP2 and AQP3 water channels in human tissue microarrays. J. Mol. Histol..

[20] Wang J.P., Hou X.H. (2007). Expression of aquaporin 8 in colonic epithelium with diarrhoea-predominant irritable bowel syndrome. Chin. Med. J..

[21] Zahn A., Moehle C., Langmann T., Ehehalt R., Autschbach F., Stremmel W., Schmitz G. (2007). Aquaporin-8 expression is reduced in ileum and induced in colon of patients with ulcerative colitis. World J. Gastroenterol..

[22] Wang K.S., Ma T., Filiz F., Verkman A.S., Bastidas J.A. (2000). Colon water transport in transgenic mice lacking aquaporin-4 water channels. Am. J. Physiol. Gastrointest Liver Physiol..

[23] Laforenza U. (2012). Water channel proteins in the gastrointestinal tract. Mol. Asp. Med..

[24] Itoh A., Tsujikawa T., Fujiyama Y., Bamba T. (2003). Enhancement of aquaporin-3 by vasoactive intestinal polypeptide in a human colonic epithelial cell line. J. Gastroenterol. Hepatol..

[25] Rai T., Sasaki S., Uchida S. (2006). Polarized trafficking of the aquaporin-3 water channel is mediated by an NH2-terminal sorting signal. Am. J. Physiol. Cell Physiol..

[26] Calamita G., Mazzone A., Bizzoca A., Cavalier A., Cassano G., Thomas D., Svelto M. (2001). Expression and immunolocalization of the aquaporin-8 water channel in rat gastrointestinal tract. Eur. J. Cell Biol..

[27] Laforenza U., Cova E., Gastaldi G., Tritto S., Grazioli M., LaRusso N.F., Splinter P.L., D’Adamo P., Tosco M., Ventura U. (2005). Aquaporin-8 is involved in water transport in isolated superficial colonocytes from rat proximal colon. J. Nutr..

[28] Laforenza U., Gastaldi G., Grazioli M., Cova E., Tritto S., Faelli A., Calamita G., Ventura U. (2005). Expression and immunolocalization of aquaporin-7 in rat gastrointestinal tract. Biol. Cell.

[29] Ikarashi N., Ushiki T., Mochizuki T., Toda T., Kudo T., Baba K., Ishii M., Ito K., Ochiai W., Sugiyama K. (2011). Effects of magnesium sulphate administration on aquaporin 3 in rat gastrointestinal tract. Biol. Pharm. Bull..

[30] Yamamoto T., Kuramoto H., Kadowaki M. (2007). Downregulation in aquaporin 4 and aquaporin 8 expression of the colon associated with the induction of allergic diarrhea in a mouse model of food allergy. Life Sci..

[31] Tsujikawa T., Itoh A., Fukunaga T., Satoh J., Yasuoka T., Fujiyama Y. (2003). Alteration of aquaporin mRNA expression after small bowel resection in the rat residual ileum and colon. J. Gastroenterol. Hepatol..

[32] Krejs G.J. (1987). VIPoma syndrome. Am. J. Med..

[33] Kane M.G., O’Dorisio T.M., Krejs G.J. (1983). Production of secretory diarrhea by intravenous infusion of vasoactive intestinal polypeptide. N. Engl. J. Med..

[34] Izzo A.A., Gaginella T.S., Capasso F. (1996). The osmotic and intrinsic mechanisms of the pharmacological laxative action of oral high doses of magnesium sulphate. Importance of the release of digestive polypeptides and nitric oxide. Magnes Res..

[35] Ikarashi N., Mochiduki T., Takasaki A., Ushiki T., Baba K., Ishii M., Kudo T., Ito K., Toda T., Ochiai W. (2011). A mechanism by which the osmotic laxative magnesium sulphate increases the intestinal aquaporin 3 expression in HT-29 cells. Life Sci..

[36] Riemann J.F., Schmidt H., Zimmermann W. (1980). The fine structure of colonic submucosal nerves in patients with chronic laxative abuse. Scand. J. Gastroenterol..

[37] Gaginella T.S., Mascolo N., Izzo A.A., Autore G., Capasso F. (1994). Nitric oxide as a mediator of bisacodyl and phenolphthalein laxative action: Induction of nitric oxide synthase. J. Pharmacol. Exp. Ther..

[38] Ikarashi N., Baba K., Ushiki T., Kon R., Mimura A., Toda T., Ishii M., Ochiai W., Sugiyama K. (2011). The laxative effect of bisacodyl is attributable to decreased aquaporin-3 expression in the colon induced by increased PGE2 secretion from macrophages. Am. J. Physiol. Gastrointest. Liver Physiol..

[39] Kuwahara M., Gu Y., Ishibashi K., Marumo F., Sasaki S. (1997). Mercury-sensitive residues and pore site in AQP3 water channel. Biochemistry.

[40] Zelenina M., Tritto S., Bondar A.A., Zelenin S., Aperia A. (2004). Copper inhibits the water and glycerol permeability of aquaporin-3. J. Biol. Chem..

[41] Ikarashi N., Kon R., Iizasa T., Suzuki N., Hiruma R., Suenaga K., Toda T., Ishii M., Hoshino M., Ochiai W. (2012). Inhibition of aquaporin-3 water channel in the colon induces diarrhea. Biol. Pharm. Bull..

[42] Mengs U., Rudolph R.L. (1993). Light and electron-microscopic changes in the colon of the guinea pig after treatment with anthranoid and non-anthranoid laxatives. Pharmacology.

[43] Hori M., Kita M., Torihashi S., Miyamoto S., Won K.J., Sato K., Ozaki H., Karaki H. (2001). Upregulation of iNOS by COX-2 in muscularis resident macrophage of rat intestine stimulated with LPS. Am. J. Physiol. Gastrointest. Liver Physiol..

[44] Lee J.Y., Cho B.J., Park T.W., Park B.E., Kim S.J., Sim S.S., Kim C.J. (2010). Dibenzylbutyrolactone lignans from Forsythia koreana fruits attenuate lipopolysaccharide-induced inducible nitric oxide synthetase and cyclooxygenase-2 expressions through activation of nuclear factor-kappab and mitogen-activated protein kinase in RAW264.7 cells. Biol. Pharm. Bull..

[45] Lehmann G.L., Carreras F.I., Soria L.R., Gradilone S.A., Marinelli R.A. (2008). LPS induces the TNF-alpha-mediated downregulation of rat liver aquaporin-8: Role in sepsis-associated cholestasis. Am. J. Physiol. Gastrointest. Liver Physiol..

[46] Horie I., Maeda M., Yokoyama S., Hisatsune A., Katsuki H., Miyata T., Isohama Y. (2009). Tumor necrosis factor-alpha decreases aquaporin-3 expression in DJM-1 keratinocytes. Biochem. Biophys. Res. Commun..

[47] Yao C., Purwanti N., Karabasil M.R., Azlina A., Javkhlan P., Hasegawa T., Akamatsu T., Hosoi T., Ozawa K., Hosoi K. (2010). Potential down-regulation of salivary gland AQP5 by LPS via cross-coupling of NF-kappaB and p-c-Jun/c-Fos. Am. J. Pathol..

[48] Zelenina M., Christensen B.M., Palmer J., Nairn A.C., Nielsen S., Aperia A. (2000). Prostaglandin E(2) interaction with AVP: Effects on AQP2 phosphorylation and distribution. Am. J. Physiol. Ren. Physiol..

[49] Nejsum L.N., Zelenina M., Aperia A., Frokiaer J., Nielsen S. (2005). Bidirectional regulation of AQP2 trafficking and recycling: Involvement of AQP2-S256 phosphorylation. Am. J. Physiol. Ren. Physiol..

[50] Kon R., Ikarashi N., Nagoya C., Takayama T., Kusunoki Y., Ishii M., Ueda H., Ochiai W., Machida Y., Sugita K. (2014). Rheinanthrone, a metabolite of sennoside A, triggers macrophage activation to decrease aquaporin-3 expression in the colon, causing the laxative effect of rhubarb extract. J. Ethnopharmacol..

[51] Zhi H., Yuan W.T. (2011). Expression of aquaporin 3, 4, and 8 in colonic mucosa of rat models with slow transit constipation. Chin. J. Gastrointest. Surg..

[52] Wang X.J., Yuan W.T., Song J.M., Zhang Z.Y. (2010). Expression and significance of aquaporin 4 in the colonic mucosa of patients with slow transit constipation. Chin. J. Gastrointest. Surg..

[53] Manara L., Bianchi G., Ferretti P., Tavani A. (1986). Inhibition of gastrointestinal transit by morphine in rats results primarily from direct drug action on gut opioid sites. J. Pharmacol. Exp. Ther..

[54] Kon R., Ikarashi N., Hayakawa A., Haga Y., Fueki A., Kusunoki Y., Tajima M., Ochiai W., Machida Y., Sugiyama K. (2015). Morphine-Induced Constipation Develops With Increased Aquaporin-3 Expression in the Colon via Increased Serotonin Secretion. Toxicol. Sci..

[55] Burks T.F., Long J.P. (1967). Release of intestinal 5-hydroxytryptamine by morphine and related agents. J. Pharmacol. Exp. Ther..

[56] Kim M., Javed N.H., Yu J.G., Christofi F., Cooke H.J. (2001). Mechanical stimulation activates Galphaq signaling pathways and 5-hydroxytryptamine release from human carcinoid BON cells. J. Clin. Investig..

[57] Iversen L. (2000). Neurotransmitter transporters: Fruitful targets for CNS drug discovery. Mol. Psychiatry.

[58] Waku T., Shiraki T., Oyama T., Maebara K., Nakamori R., Morikawa K. (2010). The nuclear receptor PPARgamma individually responds to serotonin- and fatty acid-metabolites. EMBO J..

[59] Jiang Y.J., Kim P., Lu Y.F., Feingold K.R. (2011). PPARgamma activators stimulate aquaporin 3 expression in keratinocytes/epidermis. Exp. Dermatol..

[60] Ikarashi N., Mimura A., Kon R., Iizasa T., Omodaka M., Nagoya C., Ishii M., Toda T., Ochiai W., Sugiyama K. (2012). The concomitant use of an osmotic laxative, magnesium sulphate, and a stimulant laxative, bisacodyl, does not enhance the laxative effect. Eur. J. Pharm. Sci..

